# Association between corrected serum calcium levels and 28-day mortality in patients with sepsis: A cohort study

**DOI:** 10.1097/MD.0000000000047416

**Published:** 2026-02-13

**Authors:** Manqing Li, Cong Yu

**Affiliations:** a School of Public Health, Sun Yat-sen University, Guangzhou, China; b The First Affiliated Hospital of Shenzhen University, Shenzhen Second People’s Hospital, Shenzhen University, Shenzhen, China.

**Keywords:** corrected serum calcium, eICU-CRD, ICU 28-day mortality, sepsis, U-shaped association

## Abstract

Abnormally corrected serum calcium (sCa) levels are associated with poor outcomes in various diseases, yet their relationship with short-term mortality in sepsis patients remains underexplored. This study investigates the association between corrected sCa levels and 28-day mortality in sepsis patients. We analyzed data from 7627 sepsis patients in the electronic intensive care unit Collaborative Research Database, categorizing them into 4 groups based on corrected sCa levels. Multivariate logistic regression, subgroup analysis, restricted cubic splines, and segmented regression models assessed the relationship between corrected calcium levels and mortality. Kaplan–Meier curves compared survival probabilities across groups. The 28-day mortality rate was 15.94%. After adjusting for confounders, patients in the highest corrected sCa group (>10.5 mg/dL) exhibited a significantly increased mortality risk (hazard ratio [HR]: 1.642, 95% confidence interval: 1.278–2.109) compared to the reference group (8.5–9.5 mg/dL). A U-shaped relationship was noted, with an inflection point at 9.08 mg/dL. Below this point, each 1.0 mg/dL increase in corrected sCa reduced mortality risk by 7.0% (HR, 0.930). Above 9.08 mg/dL, each 1.0 mg/dL increase raised mortality risk by 21.3% (HR, 1.213). Both low and high corrected sCa levels are linked to increased 28-day mortality in sepsis patients, highlighting the importance of monitoring calcium levels in this population.

## Graphical abstract

**Figure:**
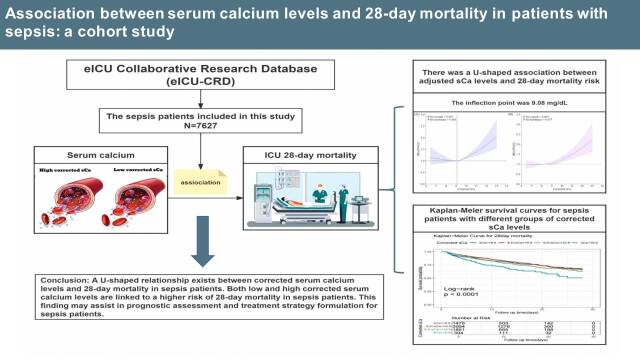

## 1. Introduction

Sepsis is defined as life-threatening organ dysfunction resulting from a dysregulated host response to infection, as established by the 3rd International Consensus Definitions for Sepsis and Septic Shock (Sepsis-3) in 2016.^[1]^ Epidemiologically, sepsis represents a significant global health challenge, exhibiting varying mortality rates across different studies and populations. In the United States, sepsis is estimated to account for 1 in every 2 to 3 deaths among hospitalized patients, with in-hospital mortality rates ranging from 12% to 26%.^[2]^ Systematic reviews indicate that septic shock, a severe manifestation of sepsis, has a mortality rate exceeding 40% in intensive care settings.^[3]^ Furthermore, the incidence of sepsis has been observed to increase over time, with the prevalence of pediatric severe sepsis rising from 3.7% to 4.4% between 2004 and 2012, resulting in approximately 11,000 deaths in the United States in 2012.^[4]^ Studies across various countries have demonstrated that sepsis is a leading cause of mortality in intensive care units (ICUs), with significant variations in incidence and outcomes based on healthcare settings and patient demographics.^[5]^ For instance, a retrospective study in Norway indicated that sepsis contributed significantly to hospital deaths, often underreported in official statistics.^[6]^ Despite the effectiveness of early antibiotic therapy in reducing 28-day mortality rates in sepsis, the overall mortality rate remains high.^[7]^ Therefore, identifying high-risk patients with poor prognoses can facilitate earlier and more effective interventions by clinicians, potentially improving mortality outcomes.

Serum calcium (sCa) levels are critical indicators of various physiological processes, playing essential roles in neuromuscular function, cardiac contractility, and bone health. Normal sCa levels typically range from 8.8 to 10.8 mg/dL, with both hypocalcemia and hypercalcemia associated with adverse health outcomes.^[8]^ Numerous studies have demonstrated a strong correlation between abnormal sCa levels and poor prognosis across various diseases. In patients with acute coronary syndrome, low sCa levels correlate with higher mortality rates, primarily due to their effects on cardiac electrophysiology.^[9]^ Similarly, in patients with acute myocardial infarction (AMI), low sCa levels are independent predictors of in-hospital mortality. In patients with chronic kidney disease (CKD), low sCa levels are linked to poor renal outcomes and increased mortality.^[10,11]^ In infectious diseases, including COVID-19, sCa levels correlate with disease severity and prognosis, with lower levels linked to worse outcomes.^[12]^ Furthermore, hypocalcemia has been identified as a marker of severity in acute pancreatitis, with significantly higher mortality rates observed in patients exhibiting low sCa levels.^[13]^

Studies suggest that hypocalcemia in sepsis may reflect cellular damage due to excessive calcium influx into cells, resulting in mitochondrial dysfunction and oxidative stress. This cellular damage can result in multiple deleterious effects, including increased apoptosis and impaired immune responses, both potentially contribute to higher mortality in sepsis patients.^[14,15]^ Additionally, the inflammatory response in sepsis is closely linked to calcium signaling pathways, where dysregulated calcium handling in immune cells may exacerbate inflammation, further complicating the clinical picture.^[16]^ A recent retrospective analysis of the Medical Information Mart for Intensive Care III database revealed a significant U-shaped association between sCa levels and 28-day mortality in sepsis patients, suggesting that both hypocalcemia and hypercalcemia are linked to increased mortality risk.^[8]^ However, evidence is lacking to guide the urgent management of sepsis patients, particularly concerning the relationship between sCa levels and short-term mortality. To address this gap, we conducted a retrospective multicenter cohort study using the electronic intensive care unit Collaborative Research Database (eICU-CRD), a comprehensive dataset from the Philips Healthcare eICU program, which includes 208 distinct ICUs across the United States from 2014 to 2015. The primary objective of this study was to determine the threshold of sCa concentrations at which mortality risk significantly escalates in sepsis patients, a crucial factor in optimizing clinical outcomes in this high-risk population.

## 2. Methods

### 2.1. Database

In this retrospective observational study, we utilized the eICU-CRD, an online international resource.^[17]^ The eICU-CRD is a publicly available, comprehensive multicenter ICU database containing detailed data from ICU admissions across the United States. The database includes high-precision data from over 200,000 ICU admissions across more than 200 hospitals between 2014 and 2015, covering demographic information, vital signs, laboratory results, medication records, and more. We completed the Collaborative Institutional Training Initiative program to obtain data access permissions, successfully passing the exam (record ID: 62969467). This process complies with the data use protocol of the PhysioNet Review Board. The Massachusetts Institute of Technology Institutional Review Board approved the study based on its retrospective design, lack of direct patient intervention, and the safe architecture certified by Privacert (Cambridge, MA) as compliant with Health Insurance Portability and Accountability Act safe harbor standards for reidentification risk. As a result, the study was granted a waiver of informed consent. The study was conducted in full accordance with the Declaration of Helsinki.

### 2.2. Study population

**Figure 1.**
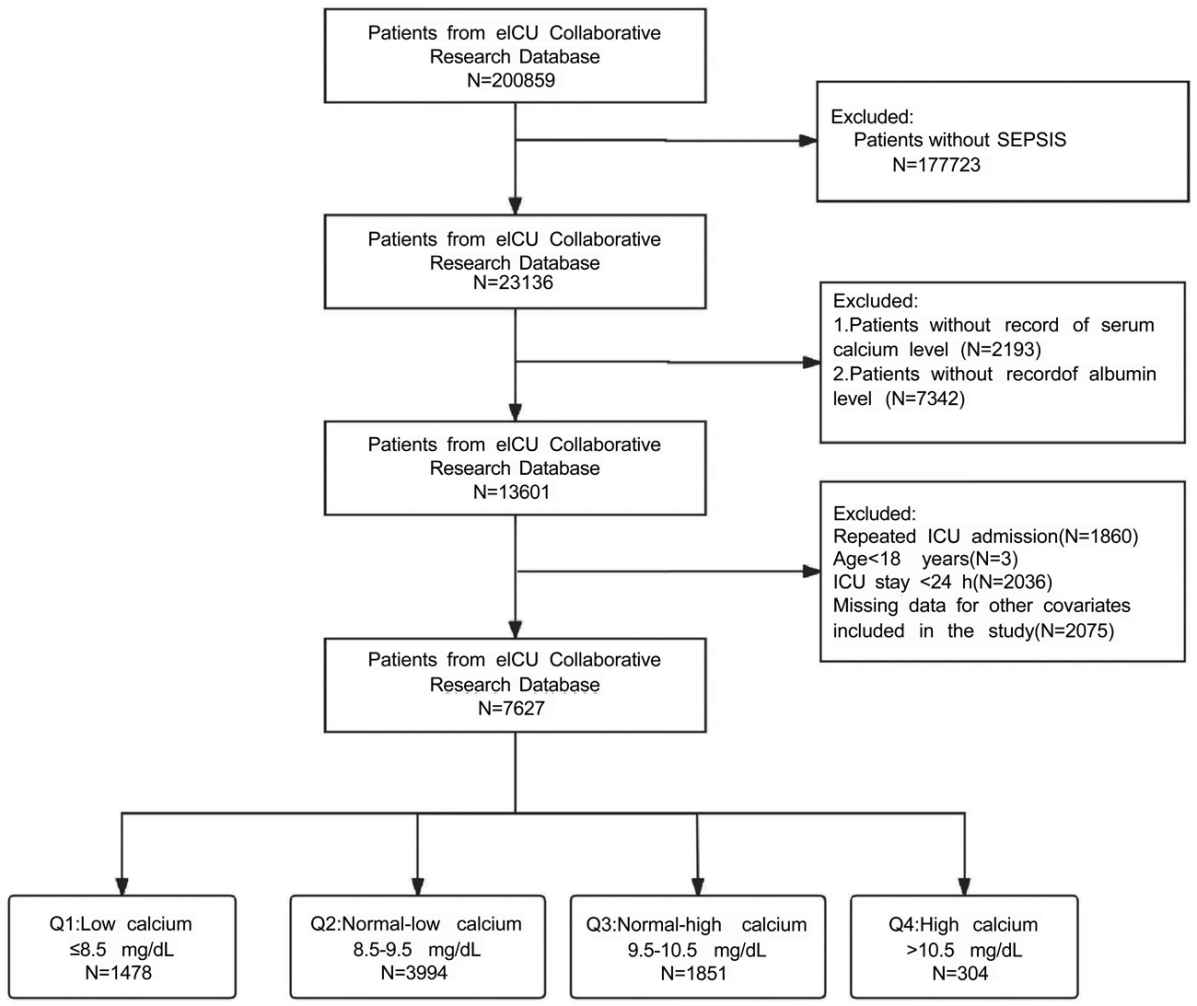
A flow chart of the inclusion and exclusion of patients. ICU = intensive care unit, SEPSIS = systemic inflammatory response syndrome.

Of the 200,859 patients identified in the eICU-CRD, 23,136 were diagnosed with sepsis. Sepsis was defined as a suspected or documented infection with an acute increase of ≥2 points in the Sequential Organ Failure Assessment score. Infections were identified using International Classification of Diseases, 9th Revision codes in the eICU-CRD. The exclusion criteria were: missing sCa data; missing albumin data; not the 1st ICU admission; ICU stay <24 hours; age <18 years; and missing data for covariates. The final analysis included 7627 sepsis patients. The study flow is depicted in Figure 1.

### 2.3. Variables

Basic demographic and clinical data for each patient were extracted from the eICU-CRD. Demographic data included age, sex, race, body mass index (BMI), and ethnicity. Medical procedures recorded included intubation status. Comorbidities included immunosuppression, cirrhosis, AMI, and diabetes mellitus (DM). Vital signs included the lowest white blood cell (WBC) count and the lowest blood sodium level. Blood biochemical indicators included red blood cell count, hemoglobin, WBC count, blood sodium, anion gap, serum potassium, albumin, and sCa. If baseline data were measured multiple times within 24 hours of ICU admission, the 1st measurement was used. Additionally, the sources of sepsis infection were recorded, including pulmonary, renal/urinary (including bladder), gastrointestinal, unknown, skin/soft tissue, other, and gynecological. To improve data reliability, sCa (mg/dL) was adjusted for albumin (g/dL) using the Payne formula: Corrected serum calcium (corrected sCa) = serum calcium + 0.8 × (4.0−albumin). The primary outcome was ICU 28-day mortality.

### 2.4. Statistical analysis

Corrected sCa was calculated for each patient and classified into 4 groups: ≤8.5, 8.5 to 9.5, 9.5 to 10.5, and >10.5 mg/dL.^[18]^ Continuous variables were reported as mean ± standard deviation or median with interquartile range, while categorical variables were expressed as counts and percentages. Differences across the quartiles of corrected sCa in the study population were compared using the Kruskal–Wallis test or 1-way analysis of variance for continuous variables, and the chi-square test or Fisher’s exact test for categorical variables.

Univariate analysis was conducted to assess the association between variables and 28-day mortality. Multivariate logistic regression models were used to evaluate the association between corrected calcium levels and 28-day mortality in sepsis patients. In model I, no covariates were adjusted. In model II, adjustments were made for covariates, including age, gender, ethnicity, and BMI. In model III, adjustments were made for age, gender, ethnicity, BMI, and potential confounding factors (intubated, dialysis, immunosuppression, cirrhosis, AMI, DM, chronic obstructive pulmonary disease (COPD), cancer, total intake, total output, use of vasopressor, use of steroid, use of antibiotics, infection organisms, WBC count, hemoglobin, serum sodium, serum potassium, serum creatinine, blood urea nitrogen and site of infection). sCa, serum calcium; HR, hazard ratio; CI, confidence interval; BMI, body mass index; AMI, acute myocardial infarction; DM, diabetes mellitus; COPD, chronic obstructive pulmonary disease; WBC, white blood cell; Ref, reference group.^[19]^ Confounders were selected based on their association with 28-day mortality (*P* < .10) or if they caused a change in the effect estimate exceeding 10%.^[20]^ Results from the multivariate analysis are presented as hazard ratios (HRs) with 95% confidence intervals (CIs) and *P* values. The restricted cubic spline (RCS) was used to explore the nonlinear relationship, and then a piecewise linear regression model was constructed to calculate the turning point. Stratified analyses and interaction tests were performed to assess whether the effect of corrected sCa levels varied across subgroups, with results displayed in a forest plot. Survival curves were generated using the Kaplan–Meier method, and the log-rank test was applied to compare survival rates across the 4 groups.

All statistical analyses were conducted using R (http://www.R-project.org, the R Foundation for Statistical Computing, Vienna, Austria) and EmpowerStats (www.empowerstats.com, X&Y Solutions, Inc., Boston). A *P* value of <.05 was considered statistically significant.

## 3. Results

### 3.1. Baseline characteristics

This study evaluated data from 6902 participants, divided into 4 groups based on corrected sCa (sCa) levels: ≤8.5, >8.5 to ≤9.5, >9.5 to ≤10.5, and >10.5 mg/dL, as shown in Table 1. The highest corrected sCa group (>10.5 mg/dL) had the highest 28-day mortality rate (26.974%), while the >8.5 to ≤9.5 mg/dL group exhibited the lowest (14.096%). Participants in the lowest corrected sCa group (≤8.5 mg/dL) were younger and had lower BMI compared to other groups. Hemoglobin and albumin levels were lowest in the ≤8.5 mg/dL group, while WBC count and anion gap were highest. In contrast, the highest corrected sCa group (>10.5 mg/dL) showed elevated potassium and WBC counts but reduced red blood cell count, hemoglobin, and albumin levels compared to other groups. Intubation rates decreased with increasing corrected sCa levels (*P* < .001), whereas the prevalence of DM increased with higher sCa levels (*P* = .002). Significant differences in sepsis infection sources were observed across groups (*P* < .001), with pulmonary infection being the most prevalent in all sCa groups. ICU 28-day mortality showed a significant association with corrected sCa levels (*P* < .001), displaying a U-shaped relationship, where both the lowest (≤8.5 mg/dL) and highest (>10.5 mg/dL) sCa groups exhibited higher mortality rates compared to the middle groups.

**Table 1:** Baseline and clinical characteristics of the study patients.

Corrected sCa	≤8.5	>8.5, ≤9.5	>9.5, ≤10.5	>10.5	*P* value
Participants	1478	3994	1851	304	
Demographics					
Age, years	62.120 ± 16.882	65.922 ± 16.016	66.691 ± 15.156	66.858 ± 13.710	<.001
Age categorical					<.001
<45	213 (14.411%)	398 (9.965%)	165 (8.914%)	18 (5.921%)	
≥45, <60	399 (26.996%)	867 (21.708%)	342 (18.476%)	67 (22.039%)	
≥60	866 (58.593%)	2729 (68.327%)	1344 (72.609%)	219 (72.039%)	
BMI, kg/m^2^	28.030 ± 8.251	28.962 ± 8.724	29.262 ± 9.176	28.360 ± 9.906	<.001
Gender, n (%)					.049
Male	755 (51.083%)	1915 (47.947%)	870 (47.002%)	159 (52.303%)	
Female	723 (48.917%)	2079 (52.053%)	981 (52.998%)	145 (47.697%)	
Ethnicity, n (%)					<.001
Caucasian	1144 (77.402%)	3172 (79.419%)	1427 (77.093%)	218 (71.711%)	
African American	108 (7.307%)	366 (9.164%)	230 (12.426%)	52 (17.105%)	
Hispanic	109 (7.375%)	200 (5.008%)	81 (4.376%)	16 (5.263%)	
Asian	57 (3.857%)	150 (3.756%)	73 (3.944%)	9 (2.961%)	
Native American	20 (1.353%)	45 (1.127%)	23 (1.243%)	4 (1.316%)	
Other/unknown	40 (2.706%)	61 (1.527%)	17 (0.918%)	5 (1.645%)	
Medical procedures					
Intubated, n (%)					<.001
No	1088 (73.613%)	3168 (79.319%)	1459 (78.822%)	248 (81.579%)	
Yes	390 (26.387%)	826 (20.681%)	392 (21.178%)	56 (18.421%)	
Dialysis, n (%)					<.001
No	1427 (96.549%)	3827 (95.819%)	1691 (91.356%)	264 (86.842%)	
Yes	51 (3.451%)	167 (4.181%)	160 (8.644%)	40 (13.158%)	
Complicating disease					
Immunosuppression, n (%)					.250
No	1377 (93.166%)	3742 (93.691%)	1754 (94.760%)	284 (93.421%)	
Yes	101 (6.834%)	252 (6.309%)	97 (5.240%)	20 (6.579%)	
Cirrhosis, n (%)					.086
No	1434 (97.023%)	3832 (95.944%)	1771 (95.678%)	287 (94.408%)	
Yes	44 (2.977%)	162 (4.056%)	80 (4.322%)	17 (5.592%)	
AMI, n (%)					.050
No	1423 (96.279%)	3855 (96.520%)	1807 (97.623%)	298 (98.026%)	
Yes	55 (3.721%)	139 (3.480%)	44 (2.377%)	6 (1.974%)	
DM, n (%)					.002
No	1354 (91.610%)	3552 (88.933%)	1642 (88.709%)	259 (85.197%)	
Yes	124 (8.390%)	442 (11.067%)	209 (11.291%)	45 (14.803%)	
COPD, n (%)					.553
No	1394 (94.317%)	3731 (93.415%)	1725 (93.193%)	286 (94.079%)	
Yes	84 (5.683%)	263 (6.585%)	126 (6.807%)	18 (5.921%)	
Cancer, n (%)					.024
No	1418 (95.940%)	3862 (96.695%)	1781 (96.218%)	284 (93.421%)	
Yes	60 (4.060%)	132 (3.305%)	70 (3.782%)	20 (6.579%)	
Blood biochemical indicators					
WBC count, cells × 109/L	15.624 ± 14.552	15.171 ± 10.469	15.781 ± 9.903	18.445 ± 13.323	<.001
Hemoglobin, g/dL	10.491 ± 2.313	10.418 ± 2.105	10.199 ± 2.134	9.881 ± 2.245	<.001
Serum sodium, mmol/L	137.898 ± 6.159	138.023 ± 6.158	138.447 ± 6.517	139.110 ± 7.390	.002
Serum potassium, mmol/L	4.038 ± 0.843	4.076 ± 0.740	4.185 ± 0.804	4.286 ± 0.832	<.001
Serum creatinine, mg/dL	2.300 ± 2.197	1.843 ± 1.627	2.145 ± 2.026	2.435 ± 2.270	<.001
Blood urea nitrogen, mg/dL	35.915 ± 26.772	33.378 ± 24.187	40.387 ± 28.532	46.645 ± 32.408	<.001
Fluid therapy					
Total intake	7510.265 ± 10,290.788	5955.791 ± 8211.328	5546.458 ± 8033.113	5567.957 ± 7943.119	<.001
Total output	3244.873 ± 4613.175	2868.846 ± 3595.686	2797.378 ± 4179.383	2402.467 ± 3004.284	<.001
Use of vasopressor, n (%)					<.001
No	1224 (82.815%)	3608 (90.336%)	1689 (91.248%)	270 (88.816%)	
Yes	254 (17.185%)	386 (9.664%)	162 (8.752%)	34 (11.184%)	
Use of steroid, n (%)					.277
No	1192 (80.650%)	3169 (79.344%)	1448 (78.228%)	248 (81.579%)	
Yes	286 (19.350%)	825 (20.656%)	403 (21.772%)	56 (18.421%)	
Use of antibiotics, n (%)					.897
No	1466 (99.188%)	3962 (99.199%)	1833 (99.028%)	302 (99.342%)	
Yes	12 (0.812%)	32 (0.801%)	18 (0.972%)	2 (0.658%)	
Infection organisms, n (%)					.752
No	1447 (97.903%)	3913 (97.972%)	1818 (98.217%)	300 (98.684%)	
Yes	31 (2.097%)	81 (2.028%)	33 (1.783%)	4 (1.316%)	
SEPSIS source of infection, n (%)					<.001
Pulmonary	473 (32.003%)	1523 (38.132%)	732 (39.546%)	122 (40.132%)	
Renal/UTI (including bladder)	336 (22.733%)	854 (21.382%)	441 (23.825%)	63 (20.724%)	
GI	281 (19.012%)	597 (14.947%)	218 (11.777%)	37 (12.171%)	
Unknown	191 (12.923%)	436 (10.916%)	181 (9.778%)	37 (12.171%)	
Cutaneous/soft tissue	81 (5.480%)	301 (7.536%)	166 (8.968%)	25 (8.224%)	
Other	113 (7.645%)	272 (6.810%)	109 (5.889%)	20 (6.579%)	
Gynecologic	3 (0.203%)	11 (0.275%)	4 (0.216%)	0 (0.000%)	
ICU 28 d mortality, n (%)					<.001
No	1238 (83.762%)	3431 (85.904%)	1520 (82.118%)	222 (73.026%)	
Yes	240 (16.238%)	563 (14.096%)	331 (17.882%)	82 (26.974%)	

Data are expressed as the mean ± SD or percentage.

AMI = acute myocardial infarction, BMI = body mass index, COPD = chronic obstructive pulmonary disease, DM = diabetes mellitus, GI = gastrointestinal, ICU = intensive care unit, sCa = serum Calcium, SD = standard deviation, SEPSIS = systemic inflammatory response syndrome, UTI = urinary tract infection, WBC = white blood cell.

### 3.2. Univariate analysis of risk factors associated with 28-day mortality in sepsis patients

Univariate Cox proportional hazards regression analysis was conducted to assess factors associated with 28-day ICU mortality. Corrected sCa levels demonstrated a significant association with mortality when analyzed both categorically and continuously. Compared to the reference group (sCa ≤ 8.5 mg/dL), patients with sCa >10.5 mg/dL had a significantly elevated mortality risk (HR: 1.642, 95% CI: 1.278–2.109, *P* < .001), while those with sCa >8.5 to ≤9.5 mg/dL showed a nonsignificant trend toward reduced risk (HR: 0.881, 95% CI: 0.757–1.025, *P* = .100), and sCa >9.5 to ≤10.5 mg/dL showed no significant association (HR: 1.067, 95% CI: 0.903–1.260, *P* = .446). As a continuous variable, each 1 mg/dL increase in sCa increased mortality risk by 11.1% (HR: 1.111, 95% CI: 1.043–1.183, *P* = .001). Age was a strong predictor, with each additional year increasing mortality risk by 2.2% (HR: 1.022, 95% CI: 1.018–1.026, *P* < .001); categorically, patients aged ≥45 to <60 years (HR: 1.842, 95% CI: 1.379–2.460, *P* < .001) and ≥60 years (HR: 2.804, 95% CI: 2.150–3.657, *P* < .001) had significantly higher risks compared to those <45 years. Gender and ethnicity showed no significant associations (*P* > .05), with female gender (HR: 1.063, 95% CI: 0.949–1.189, *P* = .292) and ethnic groups compared to Caucasians yielding HRs from 0.886 to 1.278 (all *P* > .05). Higher BMI was associated with a slight protective effect (HR: 0.984, 95% CI: 0.978–0.991, *P* < .001). Clinical factors, such as intubation (HR: 1.691, 95% CI: 1.503–1.901, *P* < .001), immunosuppression (HR: 1.452, 95% CI: 1.188–1.773, *P* < .001), cirrhosis (HR: 2.252, 95% CI: 1.832–2.769, *P* < .001), cancer (HR: 2.117, 95% CI: 1.678–2.671, *P* < .001), and vasopressor use (HR: 3.450, 95% CI: 3.054–3.898, *P* < .001) significantly increased mortality risk, while diabetes (HR: 0.769, 95% CI: 0.632–0.937, *P* = .009) and steroid use (HR: 0.821, 95% CI: 0.714–0.945, *P* = .006) were associated with reduced risk. Laboratory parameters including serum potassium (HR: 1.218, 95% CI: 1.140–1.300, *P* < .001), creatinine (HR: 1.056, 95% CI: 1.030–1.082, *P* < .001), blood urea nitrogen (HR: 1.008, 95% CI: 1.006–1.010, *P* < .001), and WBC count (HR: 1.010, 95% CI: 1.006–1.013, *P* < .001) were also significant predictors. Infection sites showed differential risks, with renal/urinary tract (HR: 0.651, 95% CI: 0.549–0.772, *P* < .001) and cutaneous/soft tissue infections (HR: 0.553, 95% CI: 0.429–0.713, *P* < .001) associated with lower mortality compared to pulmonary infections (Table 2).

**Table 2:** Univariate analysis.

	Statistics	ICU 28 d mortality	
		HR (95% CI)	*P* value
Corrected sCa			
≤8.5	1478 (19.379%)	Ref	
>8.5, ≤9.5	3994 (52.367%)	0.881 (0.757, 1.025)	.100
>9.5, ≤10.5	1851 (24.269%)	1.067 (0.903, 1.260)	.446
>10.5	304 (3.986%)	1.642 (1.278, 2.109)	<.001
Corrected sCa	9.127 + 0.815	1.111 (1.043, 1.183)	.001
Age (yr)	65.415 ± 15.973	1.022 (1.018, 1.026)	<.001
Age (yr) categorical			
<45	794 (10.410%)	Ref	
≥45, <60	1675 (21.961%)	1.842 (1.379, 2.460)	.000
≥60	5158 (67.628%)	2.804 (2.150, 3.657)	<.001
Gender			
Male	3699 (48.499%)	Ref	
Female	3928 (51.501%)	1.063 (0.949, 1.189)	.292
Ethnicity			
Caucasian	5961 (78.157%)	Ref	
African American	756 (9.912%)	0.923 (0.765, 1.113)	.402
Hispanic	406 (5.323%)	0.886 (0.682, 1.150)	.362
Asian	289 (3.789%)	0.924 (0.685, 1.246)	.602
Native American	92 (1.206%)	1.090 (0.665, 1.787)	.732
Other/unknown	123 (1.613%)	1.278 (0.859, 1.902)	.225
BMI	28.830 ± 8.806	0.984 (0.978, 0.991)	<.001
Intubated			
No	5963 (78.183%)	Ref	
Yes	1664 (21.817%)	1.691 (1.503, 1.901)	<.001
Dialysis			
No	7209 (94.519%)	Ref	
Yes	418 (5.481%)	1.117 (0.889, 1.403)	.342
Immunosuppression			
No	7157 (93.838%)	Ref	
Yes	470 (6.162%)	1.452 (1.188, 1.773)	.000
Cirrhosis			
No	7324 (96.027%)	Ref	
Yes	303 (3.973%)	2.252 (1.832, 2.769)	<.001
AMI			
No	7383 (96.801%)	Ref	
Yes	244 (3.199%)	1.086 (0.801, 1.472)	.596
DM			
No	6807 (89.249%)	Ref	
Yes	820 (10.751%)	0.769 (0.632, 0.937)	.009
COPD			
No	7136 (93.562%)	Ref	
Yes	491 (6.438%)	1.019 (0.814, 1.277)	.867
Cancer			
No	7345 (96.303%)	Ref	
Yes	282 (3.697%)	2.117 (1.678, 2.671)	<.001
Total intake	6142.225 ± 8628.839	1.000 (1.000, 1.000)	.019
Total output	2905.781 ± 3939.972	1.000 (1.000, 1.000)	<.001
Use of vasopressor			
No	6791 (89.039%)	Ref	
Yes	836 (10.961%)	3.450 (3.054, 3.898)	<.001
Use of steroids			
No	6057 (79.415%)	Ref	
Yes	1570 (20.585%)	0.821 (0.714, 0.945)	.006
Use of antibiotics			
No	7563 (99.161%)	Ref	
Yes	64 (0.839%)	0.927 (0.512, 1.678)	.802
Infection organisms			
No	7478 (98.046%)	Ref	
Yes	149 (1.954%)	1.373 (0.972, 1.941)	.072
WBC (cells × 109/L)	15.537 ± 11.387	1.010 (1.006, 1.013)	<.001
Hemoglobin (g/dL)	10.358 ± 2.164	0.990 (0.965, 1.016)	.458
Serum sodium (mmol/L)	138.145 ± 6.305	0.999 (0.991, 1.008)	.882
Serum potassium (mmol/L)	4.104 ± 0.783	1.218 (1.140, 1.300)	<.001
Serum creatinine (mg/dL)	2.028 ± 1.888	1.056 (1.030, 1.082)	.000
Blood urea nitrogen (mg/dL)	36.099 ± 26.403	1.008 (1.006, 1.010)	<.001
Site of infection			
Pulmonary	2850 (37.367%)	Ref	
Renal/UTI (including bladder)	1694 (22.211%)	0.651 (0.549, 0.772)	<.001
GI	1133 (14.855%)	0.994 (0.847, 1.167)	.941
Unknown	845 (11.079%)	1.037 (0.863, 1.247)	.698
Cutaneous/soft tissue	573 (7.513%)	0.553 (0.429, 0.713)	<.001
Other	514 (6.739%)	0.936 (0.748, 1.170)	.561
Gynecologic	18 (0.236%)	0.929 (0.299, 2.889)	.898

Data are expressed as the mean ± SD or percentage.

AMI = acute myocardial infarction, BMI = body mass index, CI = confidence interval, COPD = chronic obstructive pulmonary disease, DM = diabetes mellitus, GI = gastrointestinal, HR = hazard ratio, ICU = intensive care unit, Ref = reference, sCa = serum calcium, SD = standard deviation, UTI = urinary tract infection, WBC = white blood cell.

### 3.3. Associations of corrected sCa with 28-day mortality

The association between corrected sCa levels and 28-day mortality in sepsis patients is detailed in Table 3. When evaluated as a continuous variable, corrected sCa levels showed a significant positive correlation with 28-day mortality. In the unadjusted model I, each 1 mg/dL increase in corrected sCa was associated with an 11.1% increase in the risk of 28-day mortality (HR: 1.111, 95% CI: 1.043–1.183, *P* = .001). In model II, adjusted for age, gender, ethnicity, and BMI, the association remained statistically significant (HR: 1.092, 95% CI: 1.023–1.166, *P* = .008). In model III, which further adjusted for potential confounding factors including intubation, dialysis, immunosuppression, cirrhosis, AMI, DM, COPD, cancer, total intake, total output, use of vasopressors, steroids, antibiotics, infection organisms, WBC count, hemoglobin, serum sodium, serum potassium, serum creatinine, blood urea nitrogen, and site of infection, the association was even more pronounced (HR: 1.151, 95% CI: 1.079–1.228, *P* < .001), indicating a 15.1% increase in 28-day mortality risk per 1 mg/dL increase in corrected sCa (Table 3).

**Table 3:** Association between corrected sCa and 28-day mortality in patients with sepsis.

Exposure	Model I	Models II	Model III
	HR (95% CI) *P* value	HR (95% CI) *P* value	HR (95% CI) *P* value
Corrected sCa	1.111 (1.043, 1.183) .001	1.092 (1.023, 1.166) .008	1.151 (1.079, 1.228) <.001
Corrected sCa			
≤8.5	Ref	Ref	Ref
>8.5, ≤9.5	0.881 (0.757, 1.025) .100	0.819 (0.703, 0.953) .010	0.957 (0.819, 1.119) .584
>9.5, ≤10.5	1.067 (0.903, 1.260) .446	0.996 (0.842, 1.177) .958	1.166 (0.980, 1.387) .083
>10.5	1.642 (1.278, 2.109) <.001	1.556 (1.210, 2.002) <.001	1.693 (1.307, 2.193) <.001
*P* for trend	<.001	.007	<.001

Model I adjusted for none.

Model II adjusted for age, gender, ethnicity, and BMI.

Model III adjusted for age, gender, ethnicity, BMI, and potential confounding factors (intubated, dialysis, immunosuppression, cirrhosis, AMI, DM, COPD, cancer, total intake, total output, use of vasopressor, use of steroid, use of antibiotics, infection organisms, WBC count, hemoglobin, serum sodium, serum potassium, serum creatinine, blood urea nitrogen and site of infection).

AMI = acute myocardial infarction, BMI = body mass index, CI = confidence interval, COPD = chronic obstructive pulmonary disease, DM = diabetes mellitus, HR = hazard ratio, Ref = reference group, sCa = serum calcium, WBC = white blood cell.

**Figure 2.**
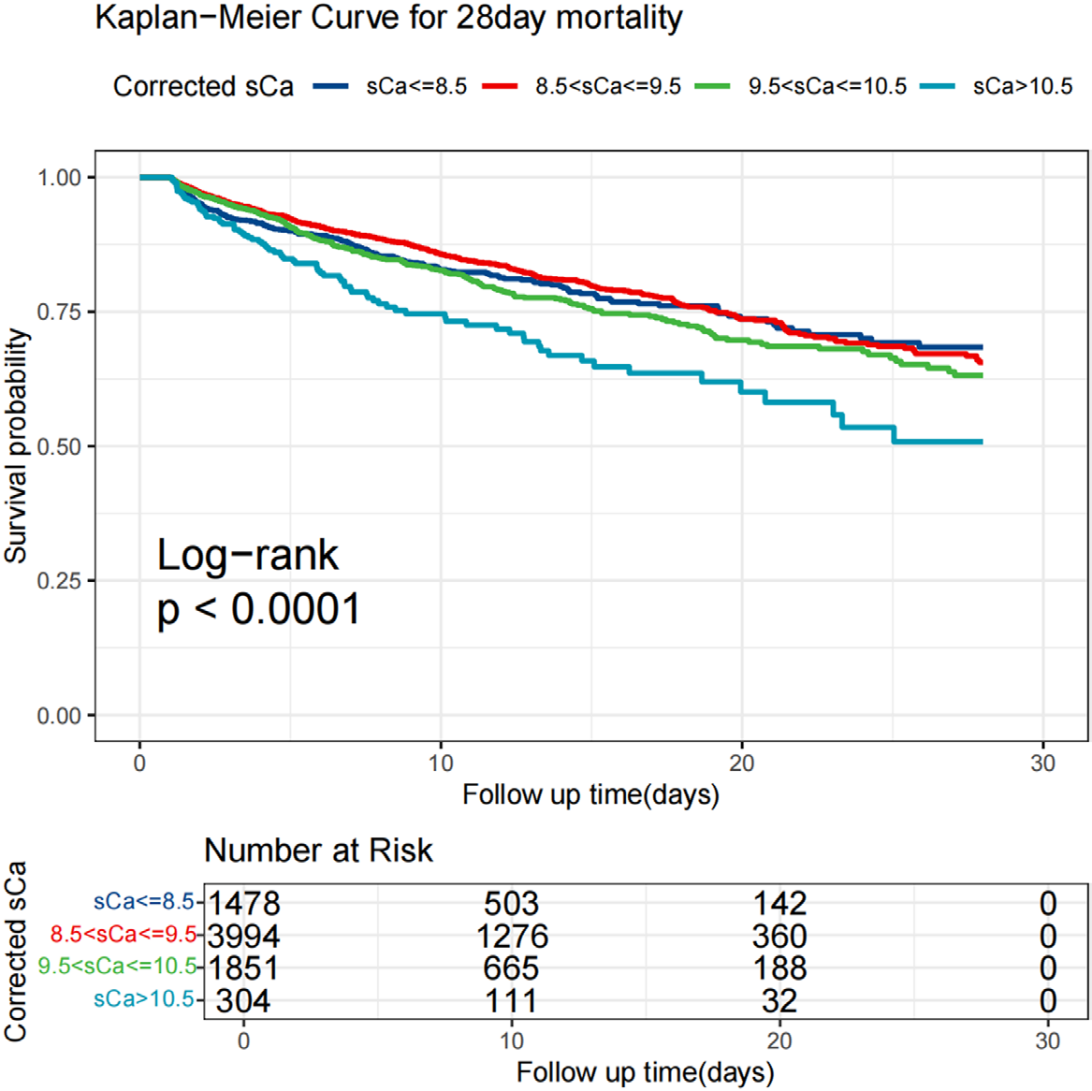
Kaplan–Meier survival curves for sepsis patients with different groups of corrected sCa levels. sCa = serum calcium.

When corrected sCa levels were categorized, using the ≤8.5 mg/dL group as the reference, the >10.5 mg/dL group exhibited the most significant increase in 28-day mortality risk. In model I, the >10.5 mg/dL group showed a 64.2% increase in mortality risk (HR: 1.642, 95% CI: 1.278–2.109, *P* < .001). This trend persisted in model II (HR: 1.556, 95% CI: 1.210–2.002, *P* < .001) and was further accentuated in the fully adjusted model III (HR: 1.693, 95% CI: 1.307–2.193, *P* < .001), demonstrating a 69.3% increase in mortality risk for the >10.5 mg/dL group. For the 8.5 to 9.5 mg/dL group, a trend towards reduced mortality risk was observed, particularly in model II (HR: 0.819, 95% CI: 0.703–0.953, *P* = .010), though this was not statistically significant in model III (HR: 0.957, 95% CI: 0.819–1.119, *P* = .584). The 9.5 to 10.5 mg/dL group showed no significant association with mortality risk across models (model III: HR: 1.166, 95% CI: 0.980–1.387, *P* = .083). Sensitivity analyses, treating corrected sCa as both 4 equal categorical variables and a continuous variable, confirmed consistent trends in the association with 28-day mortality (*P* for trend <.001 in models I and III, *P* = .007 in model II; Table 3). Among 7627 individuals, 1216 died during the follow-up period. Analysis using the Kaplan–Meier curve method indicated that the 28-day mortality survival rate of those with corrected sCa levels (>10.5 mg/dL) was significantly lower than that of those with corrected sCa levels (≤8.5 mg/dL) (*P* < .0001; Fig. 2).

### 3.4. Identification of nonlinear relationship

**Figure 3.**
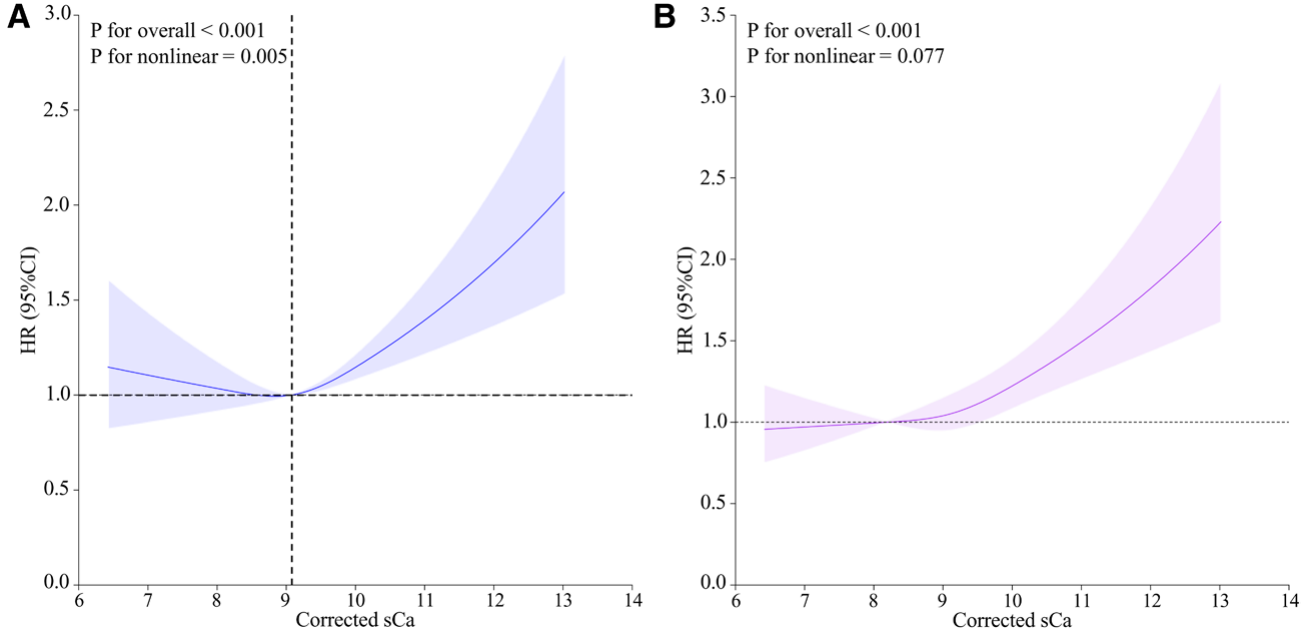
Association between sCa and 28-day mortality using an RCS model. Graphs show HRs for 28-day mortality according to corrected sCa. (A) Unadjusted. (B) Adjusted for age, gender, ethnicity, BMI, and potential confounding factors (intubated, dialysis, immunosuppression, cirrhosis, AMI, DM, COPD, cancer, total intake, total output, use of vasopressor, use of steroid, use of antibiotics, infection organisms, WBC count, hemoglobin, serum sodium, serum potassium, serum creatinine, blood urea nitrogen and site of infection). Data were fitted by an RCS Cox proportional hazards regression model, and the model was conducted with 3 knots at the 10th, 50th, and 90th percentiles of sCa. Solid lines indicate HRs, and shadow shapes indicate 95% CIs. AMI = acute myocardial infarction, BMI = body mass index, CI = confidence interval, COPD = chronic obstructive pulmonary disease, DM = diabetes mellitus, HR = hazard ratio, RCS = restricted cubic spline regression, sCa = serum calcium, WBC = white blood cell.

RCS analysis revealed a nonlinear dose-response relationship between corrected sCa levels and 28-day mortality in unadjusted models for sepsis patients (*P* for nonlinear <.05) (Fig. 3A, Table 4). When corrected sCa levels were below 9.08 mg/dL, each 1 mg/dL increase in corrected sCa was associated with a 7.0% reduction in 28-day mortality risk (HR: 0.930, 95% CI: 0.818–1.058). When corrected sCa exceeded 9.08 mg/dL; each 1 mg/dL increase was associated with a 21.3% increase in 28-day mortality risk (HR: 1.213, 95% CI: 1.123–1.311). The likelihood ratio test (*P* = .003) indicated a nonlinear association between corrected sCa levels and 28-day mortality risk in sepsis patients (Table 4). After adjusting for confounding factors, the RCS model showed no nonlinear dose-response relationship between corrected sCa and 28-day mortality risk (*P* for nonlinear >.05; Fig. 3B).

**Table 4:** Threshold effect analysis.

Model	HR (95% CI)	*P* value
Model I		
One-line slope	1.111 (1.043, 1.183)	.001
Model II		
Turning point (K)	9.08	
<9.08 slope 1	0.930 (0.818, 1.058)	.272
>9.08 slope 2	1.213 (1.123, 1.311)	<.001
LRT	.003	

Data are presented as HR (95% CI) and *P* value.

Model I, linear analysis; model II, nonlinear analysis.

Logarithmic likelihood ratio test (*P* < .05 means model II is significantly different from model I, which indicates a nonlinear association).

CI = confidence interval, HR = hazard ratio, LRT = logarithmic likelihood ratio test.

### 3.5. Association between corrected sCa and 28-day mortality in subgroups

**Figure 4.**
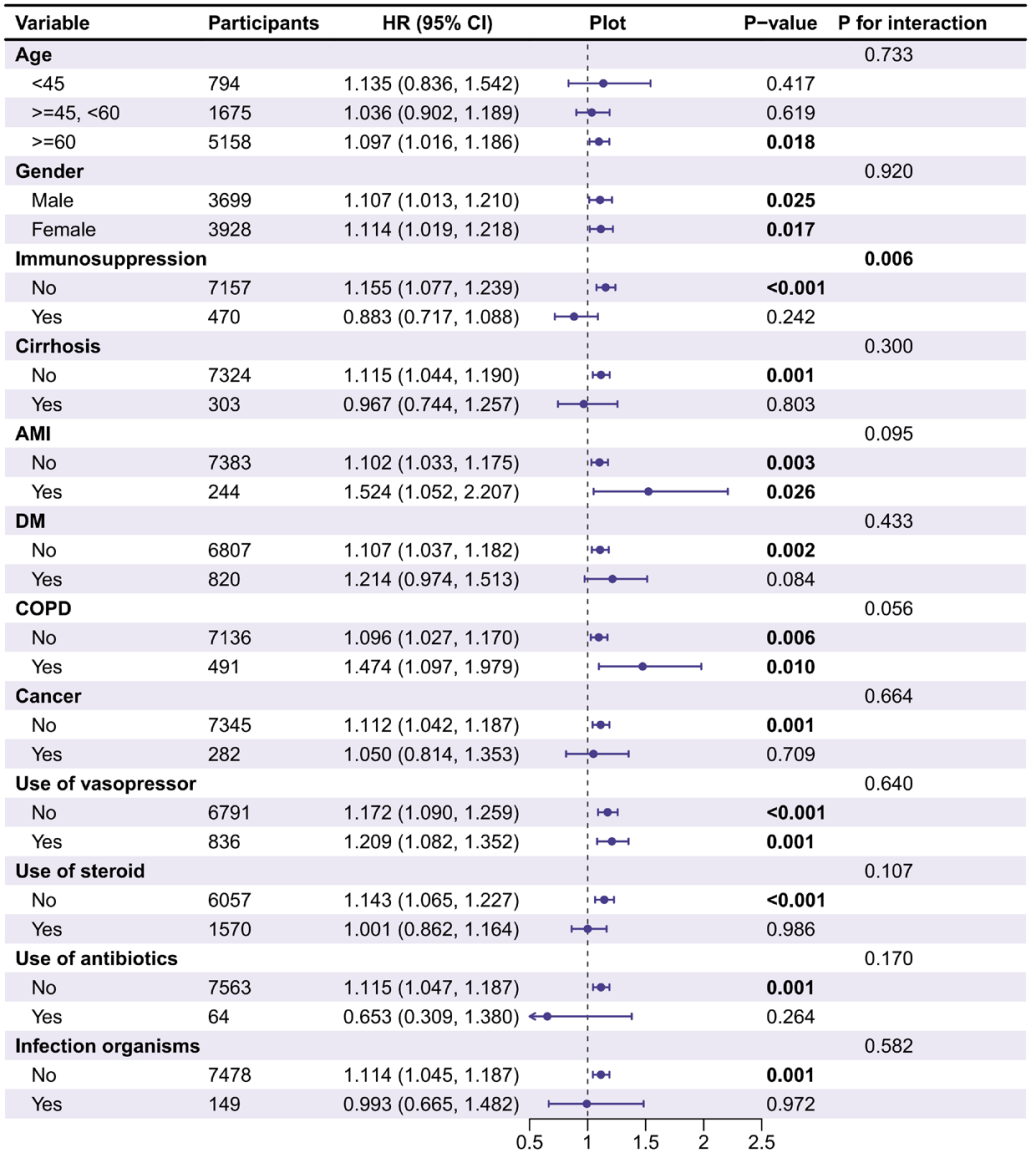
Association between corrected sCa levels and 28-day ICU mortality across subgroups. Forest plot and adjusted HRs (95% CI) for 28-day ICU mortality. 95% CI = 95% confidence interval, AMI = acute myocardial infarction; COPD = chronic obstructive pulmonary disease; DM = diabetes mellitus, HR = hazard ratio, ICU = intensive care unit.

To explore potential effect modifiers, we conducted a subgroup analysis focusing on variables likely to influence the relationship between corrected sCa levels and 28-day mortality, based on clinical relevance and prior literature. Subgroups included age, gender, immunosuppression, cirrhosis, AMI, DM, COPD, cancer, use of vasopressors, hypertension, CKD, use of antihypertensive medications, steroids, antibiotics, and infection organisms. These variables were selected due to their established associations with sepsis outcomes or calcium metabolism, as supported by studies, such as He et al and Zhang et al.^[21,22]^ Among these, a significant interaction was observed for immunosuppression (*P* for interaction = .006), indicating that the association between corrected sCa and 28-day mortality varies significantly in patients with immunosuppressive conditions. Specifically, in patients without immunosuppression (N = 7157), each 1 mg/dL increase in sCa was associated with a 15.5% increased mortality risk (HR: 1.155, 95% CI: 1.077–1.239, *P* < .001), whereas in those with immunosuppression (N = 470), the association was nonsignificant (HR: 0.883, 95% CI: 0.711–1.088, *P* = .242). This suggests that immunosuppressive conditions may modulate the impact of sCa on mortality, potentially due to altered calcium signaling pathways in immune cells, as noted in prior research.^[16]^ No significant interactions were detected for other subgroups (*P* for interaction >.05), indicating a consistent U-shaped association across most groups (Fig. 4). These findings highlight the need for tailored monitoring of sCa in immunocompromised sepsis patients to optimize clinical management.

## 4. Discussion

This retrospective cohort study used data from the eICU-CRD to investigate the association between corrected sCa levels and 28-day mortality in 7627 sepsis patients. Our analysis demonstrated a U-shaped association between corrected sCa levels and 28-day mortality risk. Patients with corrected sCa levels >10.5 mg/dL had the highest 28-day mortality rate (26.947%), while those with levels between 8.5 and 9.5 mg/dL had the lowest (14.096%). After adjusting for confounding factors, a nonlinear dose-response relationship with an inflection point at 9.08 mg/dL was identified. Below this threshold, each 1 mg/dL increase in calcium was associated with a 7.0% decrease in mortality risk, while above this threshold, each 1 mg/dL increase corresponded to a 21.3% increase in risk. Identifying this threshold has potential value for optimizing treatment, prognosis, and monitoring protocols for sepsis patients. Multiple studies have confirmed a U-shaped association between corrected sCa levels and 28-day mortality risk, consistent with observations from the eICU-CRD. In a study involving 3016 sepsis patients, using multivariate Cox regression models and smooth curve fitting, sCa levels were found to have a U-shaped association with 28-day mortality, with an inflection point at 9.0 mg/dL.^[8]^ Another large-scale study of 44,886 critically ill ICU patients reached similar conclusions, finding a U-shaped relationship between admission total serum calcium (tCa) levels and in-hospital mortality, with tCa levels outside the range of 7.6 to 9.0 mg/dL associated with higher mortality rates. However, this association disappeared after adjusting for additional clinical characteristics, suggesting that tCa may not be an independent predictor of mortality risk.^[23]^ An even larger study analyzing data from 102,245 ICU patients, using Cox proportional hazards regression and piecewise linear regression, confirmed a U-shaped relationship between albumin-corrected calcium and 30-day in-hospital mortality, with an inflection point at 8.9 mg/dL.^[24]^ These research findings consistently demonstrate a U-shaped association between blood calcium levels and short-term mortality risk in both disease-specific and general ICU patients. However, the identified inflection points differ across studies. We hypothesize that the primary reasons for these differences in inflection points are: variations in study populations; differences in adjusted variables; our data includes a larger proportion of hypercalcemia patients (Q4: corrected sCa ≥10.5 mg/dL, N = 288); and our study uses albumin-corrected sCa levels.

The mechanism underlying the U-shaped relationship between corrected sCa levels and in-hospital mortality in sepsis patients remains unclear. Several mechanisms may explain the association between low corrected sCa and higher 28-day mortality in sepsis patients: low sCa can decrease myocardial contractility, affecting the heart’s pumping function and increasing the risk of heart failure^[25]^; low sCa may worsen the inflammatory response^[26,27]^; and low calcium increases neuromuscular excitability, potentially causing muscle spasms, convulsions, or seizures, further worsening the condition.^[10,15,28]^ The mechanisms by which high corrected sCa levels lead to increased in-hospital mortality in sepsis patients include: high sCa levels may cause overexcitation of cardiomyocytes, affecting heart contractility and diastolic function, thereby increasing the risk of arrhythmias and cardiac arrest^[28,29]^; high sCa can cause metabolic disorders, including metabolic acidosis and renal dysfunction. Excessive calcium accumulation affects renal tubular function, increases urinary calcium excretion, and may lead to acute kidney injury^[25,30]^; and high sCa levels are closely associated with microangiopathy, which may lead to microvascular endothelial calcium deposition and thrombosis.^[29,31]^

Calcium supplementation may enhance the prognosis of sepsis patients through various mechanisms. Research indicates that calcium ions are essential for myocardial contraction; appropriate supplementation may enhance cardiac function, improve pumping capacity, and reduce mortality risk.^[21]^ Maintaining adequate calcium levels may bolster the immune response, enabling patients to combat infections more effectively.^[32,33]^ Additionally, calcium’s role in cellular metabolism and signal transduction indicates that supplementation may help maintain intracellular calcium homeostasis, enhance metabolic function, and reduce the risk of multiple organ dysfunction.^[24]^ Several clinical studies support this perspective, including research by Zhang et al^[22]^ and He et al,^[21]^ which found a positive correlation between calcium supplementation and survival rates in sepsis patients, particularly those with hypocalcemia. However, some studies have highlighted potential negative effects of calcium supplementation in specific contexts. For example, research by Collage et al suggests that calcium supplementation may exacerbate organ dysfunction and mortality via the calcium/calmodulin-dependent protein kinase signaling pathway.^[34]^ Therefore, while dietary calcium supplementation may be advantageous, it should be undertaken in moderation. Given these controversies, our research holds significant clinical value for managing sepsis patients. Our study is the 1st to demonstrate a U-shaped association between corrected sCa levels and 28-day mortality in sepsis patients, featuring a smooth curve and an identified inflection point (corrected sCa = 9.08 mg/dL). Clinicians can reference our research findings to formulate calcium supplementation strategies for sepsis patients, potentially reducing short-term mortality rates.

This study has several strengths: it utilized the Payne formula to adjust sCa levels for albumin, enhancing the accuracy of calcium measurements; the study population comprised critically ill patients from U.S. ICUs, with a large sample size including many individuals with abnormal sCa levels, thereby enhancing the representativeness of the data; and this study is the 1st to illustrate the relationship between corrected sCa levels and 28-day ICU mortality in sepsis patients using a smooth curve, identifying the curve’s inflection point, which holds significant clinical relevance. However, this study excluded non-sepsis patients, potentially limiting its generalizability to broader populations. Reliance on the eICU-CRD, though comprehensive, may limit generalizability due to variations in sepsis management and outcomes across global healthcare settings. Future studies using international databases are warranted. Additionally, the retrospective cohort design limits the interpretation of the association between low corrected sCa levels and increased 28-day ICU mortality as causal. Another common issue is the unavoidable presence of certain biases. Our data were derived from critically ill patients with complex conditions in U.S. ICUs. Although we adjusted for as many potential confounders as possible, unrecorded confounding factors may still have influenced the results. Therefore, further basic research and prospective studies are needed to verify the causal relationship between corrected sCa levels and 28-day mortality in sepsis patients.

## 5. Conclusion

Corrected sCa levels in sepsis patients exhibit a U-shaped association with 28-day mortality, with an inflection point at 9.08 mg/dL. Both elevated and reduced corrected sCa levels are linked to a higher risk of 28-day mortality. Therefore, corrected sCa is an independent predictor of short-term mortality in sepsis patients, facilitating the identification and early management of high-risk individuals.

## Acknowledgments

The author is very grateful to the data providers of the study.

## Author contributions

**Conceptualization:** Cong Yu.

**Funding acquisition:** Cong Yu.

**Methodology:** Manqing Li, Cong Yu.

**Data curation:** Manqing Li.

**Software:** Manqing Li.

**Visualization:** Manqing Li.

**Formal analysis:** Cong Yu.

**Investigation:** Cong Yu.

**Project administration:** Cong Yu.

**Resources:** Cong Yu.

**Writing – original draft:** Manqing Li.

**Writing – review & editing:** Cong Yu.

## References

[1] Singer M Deutschman CS Seymour CW. The third international consensus definitions for sepsis and septic shock (Sepsis-3). JAMA. 2016;315:801–10. 26903338 10.1001/jama.2016.0287 PMC4968574

[2] Donnelly JP Safford MM Shapiro NI Baddley JW Wang HE. Application of the Third International Consensus Definitions for Sepsis (Sepsis-3) classification: a retrospective population-based cohort study. Lancet Infect Dis. 2017;17:661–70. 28268067 10.1016/S1473-3099(17)30117-2 PMC5449202

[3] Vincent JL Jones G David S Olariu E Cadwell KK. Frequency and mortality of septic shock in Europe and North America: a systematic review and meta-analysis. Crit Care. 2019;23:196. 31151462 10.1186/s13054-019-2478-6 PMC6545004

[4] Paul R Melendez E Wathen B. A quality improvement collaborative for pediatric sepsis: lessons learned. Pediatr Qual Saf. 2018;3:e051. 30229187 10.1097/pq9.0000000000000051 PMC6132697

[5] Rhee C Dantes R Epstein L. Incidence and trends of sepsis in US hospitals using clinical vs claims data, 2009–2014. JAMA. 2017;318:1241–9. 28903154 10.1001/jama.2017.13836 PMC5710396

[6] Knoop ST Skrede S Langeland N Flaatten HK. Epidemiology and impact on all-cause mortality of sepsis in Norwegian hospitals: a national retrospective study. PLoS One. 2017;12:e0187990. 29149187 10.1371/journal.pone.0187990 PMC5693291

[7] Luhr R Cao Y Söderquist B Cajander S. Trends in sepsis mortality over time in randomised sepsis trials: a systematic literature review and meta-analysis of mortality in the control arm, 2002–2016. Crit Care. 2019;23:241. 31269976 10.1186/s13054-019-2528-0 PMC6610784

[8] Yan D Xie X Fu X. U-shaped association between serum calcium levels and 28-day mortality in patients with sepsis: a retrospective analysis of the MIMIC-III database. Shock. 2023;60:525–33. 37566809 10.1097/SHK.0000000000002203 PMC10581423

[9] Wang M Yan S Peng Y Shi Y Tsauo JY Chen M. Serum calcium levels correlates with coronary artery disease outcomes. Open Med (Wars). 2020;15:1128–36. 33336068 10.1515/med-2020-0154 PMC7718611

[10] Wang Y Ma H Hao X. Low serum calcium is associated with left ventricular systolic dysfunction in a Chinese population with coronary artery disease. Sci Rep. 2016;6:22283. 26924008 10.1038/srep22283 PMC4770278

[11] Lim LM Kuo HT Kuo MC. Low serum calcium is associated with poor renal outcomes in chronic kidney disease stages 3–4 patients. BMC Nephrol. 2014;15:183. 25412875 10.1186/1471-2369-15-183 PMC4255427

[12] Sun JK Zhang WH Zou L. Serum calcium as a biomarker of clinical severity and prognosis in patients with coronavirus disease 2019. Aging (Albany NY). 2020;12:11287–95. 32589164 10.18632/aging.103526 PMC7343468

[13] Chhabra P Rana SS Sharma V Sharma R Bhasin DK. Hypocalcemic tetany: a simple bedside marker of poor outcome in acute pancreatitis. Ann Gastroenterol. 2016;29:214–20. 27065735 10.20524/aog.2016.0015 PMC4805743

[14] Joseph LC Kokkinaki D Valenti MC. Inhibition of NADPH oxidase 2 (NOX2) prevents sepsis-induced cardiomyopathy by improving calcium handling and mitochondrial function. JCI Insight. 2017;2:e94248. 28878116 10.1172/jci.insight.94248 PMC5621873

[15] Liu Y Chai Y Rong Z Chen Y. Prognostic value of ionized calcium levels in neonatal sepsis. Ann Nutr Metab. 2020;76:193–200. 32756057 10.1159/000508685

[16] Zhang X Guo L Collage RD. Calcium/calmodulin-dependent protein kinase (CaMK) Iα mediates the macrophage inflammatory response to sepsis. J Leukoc Biol. 2011;90:249–61. 21372190 10.1189/jlb.0510286 PMC3133437

[17] Pollard TJ Johnson AEW Raffa JD Celi LA Mark RG Badawi O. The eICU Collaborative Research Database, a freely available multi-center database for critical care research. Sci Data. 2018;5:180178. 30204154 10.1038/sdata.2018.178 PMC6132188

[18] Fang D Chen H. Association between serum calcium level and in-hospital mortality in patients with acute myocardial infarction: a retrospective cohort study. Sci Rep. 2022;12:19954. 36402887 10.1038/s41598-022-24566-y PMC9675775

[19] Niu D Bai H Zong Y. The association between ionized calcium level and 28-day mortality in patients with sepsis: a cohort study. Sci Rep. 2025;15:22761. 40595809 10.1038/s41598-025-05090-1 PMC12216518

[20] Jaddoe VWV De Jonge LL Hofman A Franco OH Steegers EAP Gaillard R. First trimester fetal growth restriction and cardiovascular risk factors in school age children: population based cohort study. BMJ. 2014;348:g14. 24458585 10.1136/bmj.g14 PMC3901421

[21] He W Huang L Luo H. The positive and negative effects of calcium supplementation on mortality in septic ICU patients depend on disease severity: a retrospective study from the MIMIC-III. Crit Care Res Pract. 2022;2022:1–12. 10.1155/2022/2520695 PMC9242801 35782335

[22] Zhang Z Chen K Ni H. Calcium supplementation improves clinical outcome in intensive care unit patients: a propensity score matched analysis of a large clinical database MIMIC-II. Springerplus. 2015;4:594. 26543729 10.1186/s40064-015-1387-7 PMC4627965

[23] Wang B Gong Y Ying B Cheng B. Association of initial serum total calcium concentration with mortality in critical illness. Biomed Res Int. 2018;2018:1–8. 10.1155/2018/7648506 PMC6038688 30046608

[24] Qin X Cen J Hu H. Non-linear relationship between albumin-corrected calcium and 30-day in-hospital mortality in ICU patients: a multicenter retrospective cohort study. Front Endocrinol. 2022;13:1059201. 10.3389/fendo.2022.1059201 PMC9810799 36619536

[25] Liang Y Zhao L Huang J Wu Y. A nomogram to predict 28-day mortality in neonates with sepsis: a retrospective study based on the MIMIC-III database. Transl Pediatr. 2023;12:1690–706. 37814720 10.21037/tp-23-150 PMC10560361

[26] D’Elia JA Weinrauch LA. Calcium ion channels: roles in infection and sepsis mechanisms of calcium channel blocker benefits in immunocompromised patients at risk for infection. Int J Mol Sci. 2018;19:2465. 30134544 10.3390/ijms19092465 PMC6164603

[27] Rattis BAC Freitas AC Oliveira JF. Effect of verapamil, an L-type calcium channel inhibitor, on caveolin-3 expression in septic mouse hearts. Oxid Med Cell Longev. 2021;2021:6667074. 33927797 10.1155/2021/6667074 PMC8052133

[28] Lutsey PL Alonso A Michos ED. Serum magnesium, phosphorus, and calcium are associated with risk of incident heart failure: the Atherosclerosis Risk in Communities (ARIC) Study. Am J Clin Nutr. 2014;100:756–64. 25030784 10.3945/ajcn.114.085167 PMC4135486

[29] Foley RN Collins AJ Ishani A Kalra PA. Calcium-phosphate levels and cardiovascular disease in community-dwelling adults: the Atherosclerosis Risk in Communities (ARIC) Study. Am Heart J. 2008;156:556–63. 18760141 10.1016/j.ahj.2008.05.016

[30] Zheng X Li Y Cheng Q Wang L. Predictive value of ionized calcium for prognosis of sepsis in very low birth weight infants. J Inflamm Res. 2022;15:3749–60. 35799618 10.2147/JIR.S369431 PMC9255904

[31] Reid IR Bolland MJ. Does widespread calcium supplementation pose cardiovascular risk? Yes: the potential risk is a concern. Am Fam Physician. 2013;87:Online. 23418770

[32] Luschini MA Fletcher DJ Schoeffler GL. Retrospective study: incidence of ionized hypocalcemia in septic dogs and its association with morbidity and mortality: 58 cases (2006–2007). J Vet Emerg Crit Care (San Antonio). 2010;20:406–12. 20731806 10.1111/j.1476-4431.2010.00553.x

[33] Sood A Singh G Singh TG Gupta K. Pathological role of the calcium-sensing receptor in sepsis-induced hypotensive shock: therapeutic possibilities and unanswered questions. Drug Dev Res. 2022;83:1241–5. 35689439 10.1002/ddr.21959

[34] Collage RD Howell GM Zhang X. Calcium supplementation during sepsis exacerbates organ failure and mortality via calcium/calmodulin-dependent protein kinase kinase signaling. Crit Care Med. 2013;41:e352–60. 23887235 10.1097/CCM.0b013e31828cf436 PMC3812408

